# Data on assessment of physical, chemical and biological characteristics of effluent from wastewater treatment in Torbat Heydarieh, Iran

**DOI:** 10.1016/j.dib.2018.05.086

**Published:** 2018-06-01

**Authors:** Elham AlSadat Heidari, Hosein Alidadi, Ali Asghar Najafpoor, Seyed Mohsen Mohseni, Aliakbar Dehghan, Ali Sheibani, Maryam Sarkhosh

**Affiliations:** a Student Research Committee, Department of Environmental Health Engineering, School of Health, Mashhad University of Medical Sciences, Mashhad, Iran; b Social Determinants of Health Research Center, Mashhad University of Medical Sciences, Mashhad, Iran; c Student Research Committee, Department of Environmental Health Engineering, School of Health, Mashhad University of Medical Sciences, Mashhad, Iran; d Student Research Committee, Department of Environmental Health Engineering, School of Health, Shahid Beheshti University of Medical Sciences, Tehran, Iran

**Keywords:** Heavy metals, Effluent, Irrigation, Influent, Standard

## Abstract

Data on the chemical, physical and biological of effluent from wastewater treatment are provided in table format in the current article. Samples were taken in Peak Flows at effluent Treatment Plants. Sampling and tests were conducted according to the standards methods. The collected data were analyzed by SPSS software and excel program. Nickel metal showed higher amounts than the standards required for irrigation agricultural land. Data could be useful from environmental and agricultural sciences to those concerned about heavy metals, Alkalinity, EC, COD, BOD_5_ and Microbial concentrations threats.

**Specifications Table**

**Table:** 

Subject area	*Environmental Sciences*
More specific subject area	Pollutants in effluents
Type of data	*Figure and table*
How data was acquired	*Heavy Metals measured with Flame Photometer. TSS was measured by drying oven. Electrical conductivity (EC), and pH of samples were measured by the portable pH.EC.TDS Meter of Hanna instruments. Electrochemical probes were used for DO measuring. BOD measurement was carried out with a manometer instrument. Total and fecal coliform was measured with membrane filtration technique.*
Data format	*Raw, analyzed.*
Experimental factors	*The data were obtained in 2016. All effluent samples in polyethylene bottles were stored in a dark place at 4 °C temperature until the analysis.*
Experimental features	*The mentioned parameters above, were analyzed according to the standards for water and wastewater treatment handbook and compared with standard.*
Data source location	*Torbat Heydarieh, Iran*
Data accessibility	*The data are available within this paper.*

**Value of the data**

● Due to limited data in the area, the data can help to better understand the irrigation water quality in the area and provide further studies.
● Crops irrigation with wastewater treatment effluent can take risks on human health as consumers.
● The data shown here can be used for health risk assessment of pollutants for effluent disposal.

## 1 Data

**Fig. 1:**
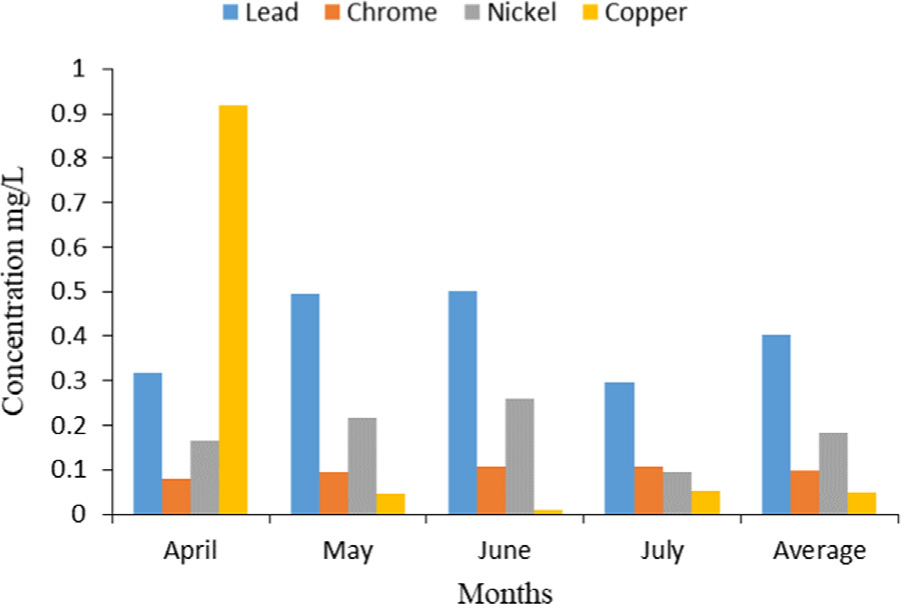
The average concentrations of each heavy metal in each sampling month.

Crops irrigation with wastewater treatment effluent can take risks on human health as consumers. It is due to absorption and accumulation of heavy metals. A summary of effluent and influent quality characteristics are presented in Table 1. The data of heavy metals measurement in wastewater treatment effluent has been shown in Table 2 and Fig. 1. Also they are WHO, EPA, and department of environment of Iran standards about acceptable heavy metals concentration wastewater treatment effluent for agriculture fields irrigation has been shown in Table 2 [1]. The data comparing with WHO, EPA, and department of environment of Iran standards in Table 2 show the concentration measurement of are more than WHO and EPA standards, but they corresponded with department of environment of Iran standards. In Table 3, total and fecal coliform nematode eggs in effluent are shown.

**Table 1:** Characteristics wastewater treatment effluent and influent.

**Parameter**	**Units**	**Influent**	**Effluent**	**Standards for discharge to surface**	**Standards for agricultural use**
**pH**	–	7.6±0.5	7.8±0.2	6.5–8.5	6.5–8.4
**DO**	mg/L	1.1±0.4	2.5±0.6	2	2
**Alkalinity**	mg CaCO_3_/L	235±24	150±15		
**TSS**	mg/L	115±18	38±3	40	100
**EC**	ds/m	0.13±0.02	0.113±0.009	–	2.97
**COD**	mg/L	304.5±18.7	58.1±6.2	60	200
**BOD**_**5**_	mg/L	121.6±27.6	24.3±3.7	30	100

**Table 2:** Heavy metals concentration in wastewater treatment effluent and compare with different standards.

**Parameter**	**Units**	**Effluent**	**Iran Standards for agricultural use**	**EPA Standards for agricultural use**	**WHO Standards for agricultural use**
**Ni**	µg/L	184±96	2000	200	200
**Pb**	µg/L	402±80	1000	5000	5000
**Cu**	µg/L	50±62	200	200	200
**Cr**	µg/L	98±12	1000	100	100

**Table 3:** Microbial concentrations in wastewater treatment effluent.

**Parameter**	**Units**	**Effluent**	**Standards for discharge to surface**	**Standards for agricultural use**
**Total Coliform**	MPN/100 mL	310±55	1000	1000
**Fecal Coliform**	MPN/100 mL	1320±37	400	400
**Nematode eggs**	Number/L	0	–	1>

## 2 Experimental design, materials and methods

### 2.1 Area description

**Fig. 2:**
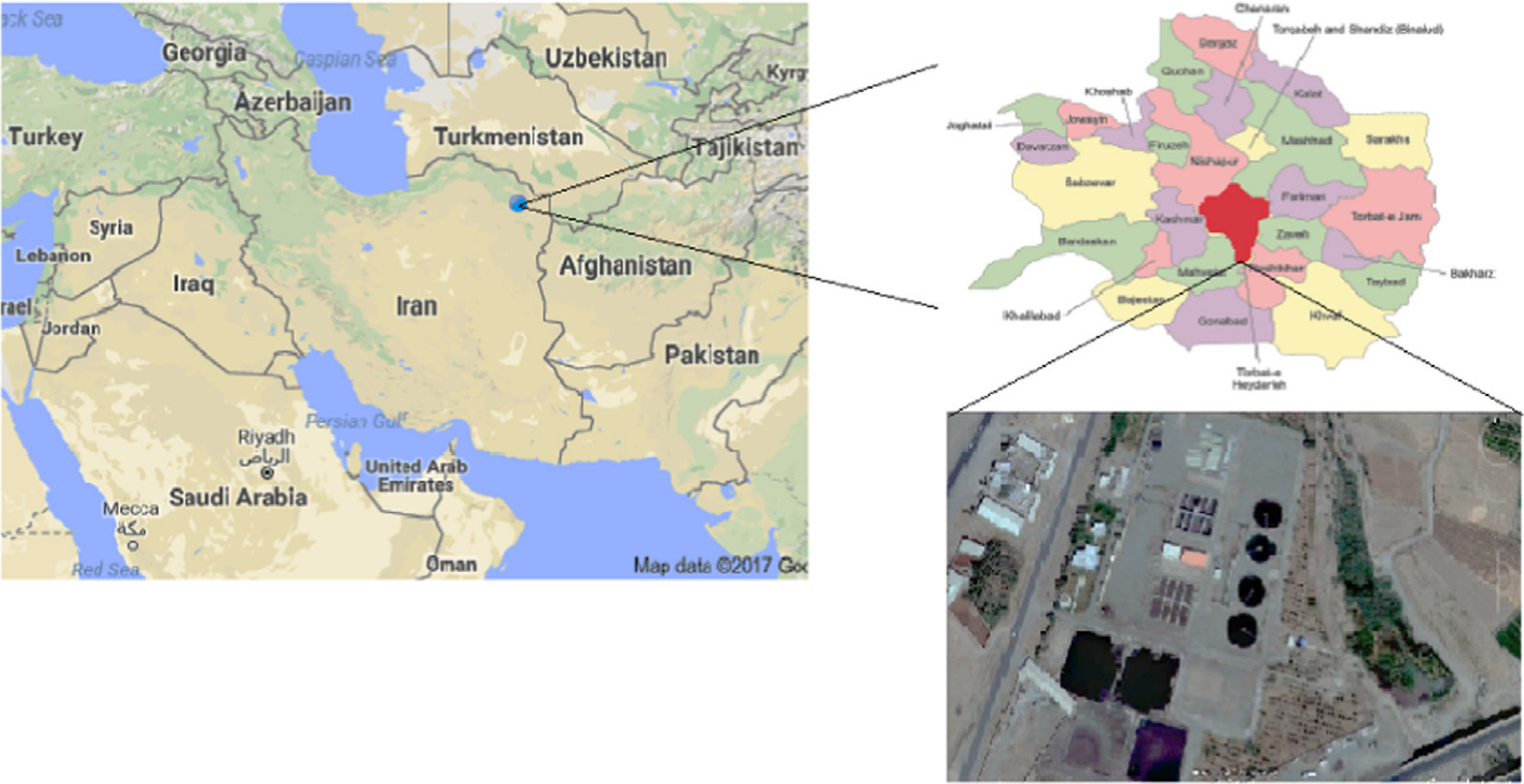
Location of wastewater treatment plant.

Torbat Heydarieh province has a 23.888 km^2^ area and locates in southwest of Mashhad (capital of state). It has a 142 km distance with Mashhad. It locates in the longitude 59° and 12 min east and the latitude 34 degree and 17 min north. Population of city is 267604 according to last census in 2006 [2]. The wastewater treatment of Torbat Heydarieh locates in southwest of city. The biological reactor of wastewater treatment is extended activated sludge. Per capita water consumption almost calculated 216 Lpcd and it is estimated to decrease 264 Lpcd on 2020. Location of Torbat Heydarie province and wastewater treatment showed in Fig. 2.

### 2.2 Sample collection and analytical procedures

This research conducted on wastewater treatment plant of Torbat Heydarieh province for four months from April to July 2016. Samples were taken in peak hours and transported to laboratory for experimenting under standard conditions. Samplings and experiments took according to the standard methods for water and wastewater treatment handbook [3], [4].

## Appendix A Supplementary materials

The following KMZ files contain the Google maps of the most important areas described in this article.

Map KMZ file containing the Google map.
