# Supplementary figures and images for: Data on assessment of physical, chemical and biological characteristics of effluent from wastewater treatment in Torbat Heydarieh, Iran
Source: Data Brief. 2018 Jun 1;19:1287–90. doi: 10.1016/j.dib.2018.05.086 (PMC6139534; doi:10.1016/j.dib.2018.05.086)

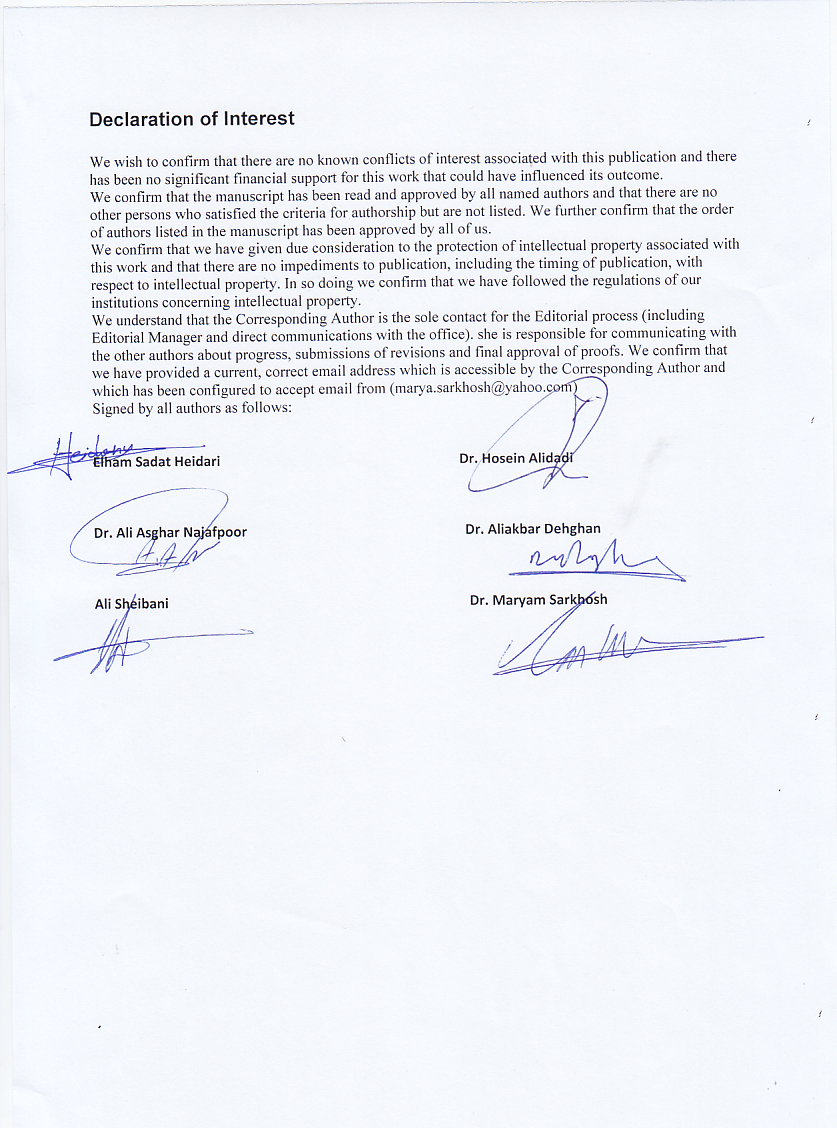

Supplement: Supplementary file 1 — Supplementary material [file mmc1.zip › sc0001.bmp]
